# A Highly Selective Turn-on and Reversible Fluorescent Chemosensor for Al^3+^ Detection Based on Novel Salicylidene Schiff Base-Terminated PEG in Pure Aqueous Solution

**DOI:** 10.3390/polym11040573

**Published:** 2019-03-27

**Authors:** Liping Bai, Yuhang Xu, Guang Li, Shuhui Tian, Leixuan Li, Farong Tao, Aixia Deng, Shuangshuang Wang, Liping Wang

**Affiliations:** School of Materials Science and Engineering, Liaocheng University, Liaocheng 252059, China; lppl131401@163.com (L.B.); 17865813297@163.com (Y.X.); ajunahui@163.com (S.T.); lileixuan6@163.com (L.L.); taofarong@lcu.edu.cn (F.T.); dengaixia@lcu.edu.cn (A.D.); wangshuangshuang@lcu.edu.cn (S.W.)

**Keywords:** aluminum ions, fluorescent chemosensor, schiff base, water-soluble polymer, test strips

## Abstract

The development of highly selective and sensitive chemosensors for Al^3+^ detection in pure aqueous solution is still a significant challenge. In this work, a novel water-soluble polymer PEGBAB based on salicylidene Schiff base has been designed and synthesized as a turn-on fluorescent chemosensor for the detection of Al^3+^ in 100% aqueous solution. PEGBAB exhibited high sensitivity and selectivity to Al^3+^ over other competitive metal ions with the detection limit as low as 4.05 × 10^−9^ M. PEGBAB displayed high selectivity to Al^3+^ in the pH range of 5–10. The fluorescence response of PEGBAB to Al^3+^ was reversible in the presence of ethylenediaminetetraacetic acid (EDTA). Based on the fluorescence response, an INHIBIT logic gate was constructed with Al^3+^ and EDTA as two inputs. Moreover, test strips based on PEGBAB were fabricated facilely for convenient on-site detection of Al^3+^.

## 1. Introduction

Aluminum, the most abundant metal element in the earth’s crust, has been widely applied in industry and daily life [1,2]. However, as a non-essential element for living systems, the frequent use of aluminum-containing products can lead to overloading of Al^3+^ in the living body, which may cause Al-related bone disease and various neurodegenerative diseases such as dialysis encephalopathy, amyotrophic lateral sclerosis, Alzheimer’s disease, and Parkinson’s disease [3,4,5,6]. The World Health Organization (WHO) recommended that the daily intake of aluminum should be less than 3–10 mg per day [7]. Furthermore, the high concentration of Al^3+^ is also harmful to growing plants and aquatic life. Considering the harmful effect of Al^3+^ on human health and environment, it is quite urgent to develop efficient approaches for Al^3+^ detection with high selectivity and sensitivity. So far, a series of common methods have been used to detect Al^3+^ ions, such as atomic absorption spectroscopy, graphite furnace atomic absorption spectrometry, inductively-coupled plasma atomic emission spectrometry, inductively-coupled plasma mass spectrometry, and voltammetry [8,9,10,11,12,13]. However, these methods are expensive, relatively complex and time-consuming in practice. Compared with these common methods, fluorescent chemosensors have attracted significant attention in recent years due to their operational simplicity, low cost, naked eye detection, real-time monitoring, time saving, high selectivity, and sensitivity [14,15,16,17,18,19,20,21,22,23,24]. However, the development of fluorescent chemosensors for Al^3+^ is relatively lagging behind because of its strong hydration ability in water, poor coordination ability and the lack of suitable spectroscopic characteristics [25,26,27].

Owing to the individual photophysical properties and the strong binding abilities to various metal ions, Schiff base derivatives have been extensively employed to construct fluorescent chemosensors for selective detection of metal ions [28,29,30,31,32,33,34,35,36,37,38,39,40]. To the best of our knowledge, most Schiff base-based fluorescent chemosensors are small molecular derivatives which cannot be dissolved in 100% water and have to be performed in the mixed aqueous solution with an organic cosolvent for the detection of metal ions. The poor water solubility restricts their practical applications to some extent [41,42]. Therefore, the development of Schiff base-based chemosensors with excellent water solubility is necessary and significant. Nowadays, polymer-based fluorescent chemosensors have attracted a wide range of interests due to their simplicity of use, signal amplification, combination of different outputs, and ease of fabrication into devices [43,44]. Immobilizing small molecular receptors onto hydrophilic polymers has been proved to be an elegant strategy to construct water-soluble chemosensors [45,46]. Therefore, the combination of Schiff base derivatives and hydrophilic polymers could be an efficient pathway to develop fluorescent chemosensors for selective detection of Al^3+^ in 100% aqueous solution.

**Scheme 1:**
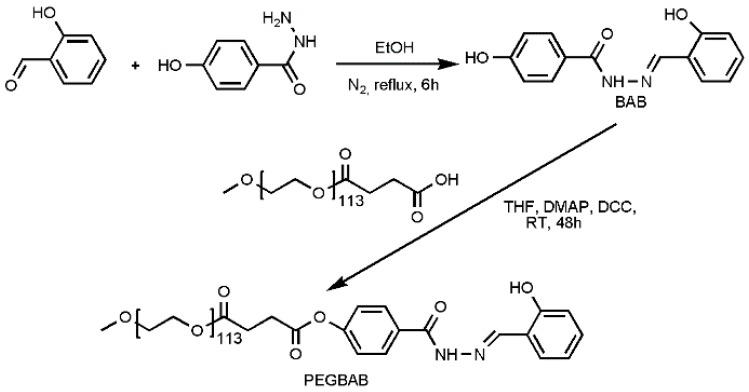
Synthetic route of chemosensor PEGBAB.

Encouraged by these studies and in continuation to the relative work of water-soluble polymer chemosensors in our group [47,48,49,50,51], we have successfully designed and synthesized a salicylidene Schiff base-terminated water-soluble polymer PEGBAB (Scheme 1). PEGBAB exhibited excellent water solubility in 100% water, and could serve as an efficient turn-on fluorescent chemosensor for Al^3+^ in pure aqueous solution. The detection limit was calculated to be 4.05 × 10^−9^ M, meaning the ultrasensitive fluorescence response of PEGBAB to Al^3+^. PEGBAB possessed high selectivity to Al^3+^ with strong anti-interference ability and showed excellent fluorescence response to Al^3+^ over a wide pH range. An INHIBIT logic gate was constructed based on the reversible sensing property of PEGBAB to Al^3+^. Moreover, test strips coated with PEGBAB have been fabricated and applied to detect Al^3+^ in practical samples.

## 2. Materials and Methods 

### 2.1. Materials

Salicylaldehyde, 4-hydroxybenzhydrazide, the nitrate or chloride salts of various metal ions (Al^3+^, Ba^2+^, Ce^3+^, Cd^2+^, Co^2+^, Cr^3+^, Cu^2+^, Fe^3+^, Hg^2+^, In^3+^, K^+^, Mn^2+^, Na^+^, Ni^2+^, Pb^2+^, and Zn^2+^) and all other chemicals were obtained from Sinopharm Chemical Reagent Co., Ltd. (Shanghai, China) and used as received. Polyethylene glycol monomethyl ether (PEG, *M*_n_ = 5000) was purchased from Sigma-Aldrich. Deionized water was used throughout the whole experiment process. Carboxylated PEG was synthesized according to the literature procedure [52]. The filter papers for the preparation of test strips were provided by Hangzhou Special Paper Industry Co., Ltd. (Hangzhou, China).

### 2.2. Characterization

^1^H NMR (400 MHz) and ^13^C NMR (100 MHz) spectra were measured by a Varian Mercury 400MHz spectrometer (Palo Alto, CA, USA) with DMSO-*d_6_* or CDCl_3_ as the solvent and TMS as the internal standard. The ESI-MS spectrum was recorded on a LTQ Orbitrap mass spectrometer (Thermo Fisher Scientific, Breman, Germany). UV-Vis absorption spectra were recorded on a Shimadzu UV-3600 spectrophotometer (Tokyo, Japan). Fluorescence spectra were measured using a Hitachi F-7000 fluorescence spectrophotometer (Tokyo, Japan). The pH values were measured by a Mettler-Toledo FE20 pH meter (Shanghai, China).

### 2.3. Synthesis of Salicylidene Schiff Base Derivative BAB

Salicylaldehyde (1.1 mL, 10.54 mmol) and 4-hydroxybenzhydrazide (0.8g, 5.26 mmol) were dissolved in 20 mL of ethanol. The reaction mixture was refluxed for 6 h with stirring under nitrogen atmosphere. After completion of the reaction, the resulting precipitate was filtered and washed three times with cold ethanol. Then, a white powder product BAB was obtained by drying under vacuum at room temperature for 24 h (87% yield). ^1^H NMR (400MHz, DMSO-*d_6_*) δ (ppm): 11.93 (s, 1H), 11.43 (s, 1H), 10.19 (s, 1H), 8.60 (s, 1H), 7.84 (d, *J* = 8.6 Hz, 2H), 7.52 (dd, *J* = 7.6, 1.4 Hz, 1H), 7.29 (t, *J* = 7.8 Hz, 1H), 6.87-6.94 (m, 4H); 13C NMR (100MHz, DMSO-*d*_6_) δ (ppm): 162.47, 160.96, 157.47, 147.63, 131.17, 129.77, 129.66, 123.21, 119.31, 118.72, 116.43, 115.16; ESI-MS (*m*/*z*): calculated for C_14_H_13_N_2_O_3_ [M + H]^+^: 257.0921; found: 257.0918.

### 2.4. Synthesis of Chemosensor PEGBAB

Carboxylated PEG (0.8 g, 0.157 mmol) and BAB (0.041 g, 0.160 mmol) were dried by azeotropic distillation in toluene for 2 h. After the removal of toluene by reduced pressure distillation, the obtained samples were dissolved in 10 mL dry THF. And then, 4-(dimethylamino) pyridine (10.0 mg) and *N*,*N*’-dicyclohexylcarbodiimide (43.0 mg, 0.21 mmol) were added rapidly. The mixed solution was stirred for 48 h at room temperature. After filtration, the filtrate was concentrated by reduced pressure distillation and precipitated in cold diethyl ether three times. The white powder product PEGBAB was collected by filtration under reduced pressure and dried under vacuum at room temperature for 24 h (86% yield).

### 2.5. Preparation of Test Solutions for Spectroscopic Measurements

The stock solutions of PEGBAB (1 × 10^−4^ M) and various metal ions (0.015 M) were prepared with deionized water as solvent, respectively. Three milliliters of measurement solutions with the required concentration of PEGBAB and metal ions were made by mixing the appropriate amount of the stock solutions and deionized water. The pH values of the test solutions were adjusted by adding the appropriate amount of HCl or NaOH. The UV-Vis and fluorescence spectra of the solutions were recorded at room temperature.

### 2.6. Application as Test Strips

PEGBAB (50 mg) was dissolved in methanol (10 mL) as the stock solution. The filter papers were immersed into the stock solution for 5 min and then dried in air to obtain test strips. The solutions of various metal ions (100 μM) were prepared with deionized water as a solvent. Three solutions of Al^3+^ (100 μM) were prepared in different water environments including deionized water, tap water and river water (Tuhai River). After being dipped in the solutions of different metal ions for 5 s, the test strips were taken out and observed under a 365 nm UV lamp.

## 3. Results and Discussion

### 3.1. Synthesis of PEGBAB

The synthetic route of the water-soluble chemosensor PEGBAB is shown in Scheme 1. Salicylidene Schiff base derivative BAB was synthesized by the typical Schiff base condensation reaction between salicylaldehyde and hydroxybenzhydrazide in ethanol. The structure of BAB was characterized by ^1^H NMR, ^13^C NMR and ESI-MS spectra (Figures S1–S3). BAB exhibited poor solubility in 100% water, which was the same as most of the small-molecule Schiff base derivatives. And so the hydrophilic polymer PEG was chosen as water-soluble carrier to achieve the water solubility of BAB. Then Schiff base derivative BAB-terminated polymer PEGBAB was obtained by the esterification of carboxylated PEG with BAB at room temperature. The successful synthesis of PEGBAB was confirmed by ^1^H NMR and ^13^C NMR analysis (Figures S4 and S5). Compared with the poor water solubility of BAB, PEGBAB could be dissolved directly in 100% water.

### 3.2. UV-Vis and Fluorescence Studies

The photophysical properties of PEGBAB were investigated by UV-Vis absorption and fluorescence response of aqueous solution of PEGBAB (10 μM) toward various metal ions, such as Al^3+^, Ba^2+^, Ce^3+^, Cd^2+^, Co^2+^, Cr^3+^, Cu^2+^, Fe^3+^, Hg^2+^, In^3+^, K^+^, Mn^2+^, Na^+^, Ni^2+^, Pb^2+^ and Zn^2+^. Initially, the sensing behavior of PEGBAB with various metal ions in pure aqueous solution was examined by UV-Vis absorption spectroscopy. As shown in Figure S6, new bands in the region between 350 nm and 420 nm were observed after the addition of 2 equiv. of Al^3+^, Cu^2+^ and Ni^2+^ into PEGBAB solutions, while other metal ions caused ignorable changes. Although Cu^2+^ and Ni^2+^ could enhance somewhat the absorbance intensity at 370 nm, the responses were weaker compared to the Al^3+^ case. Therefore, PEGBAB can be utilized to selectively detect Al^3+^ among various metal ions in pure aqueous solution.

**Figure 1:**
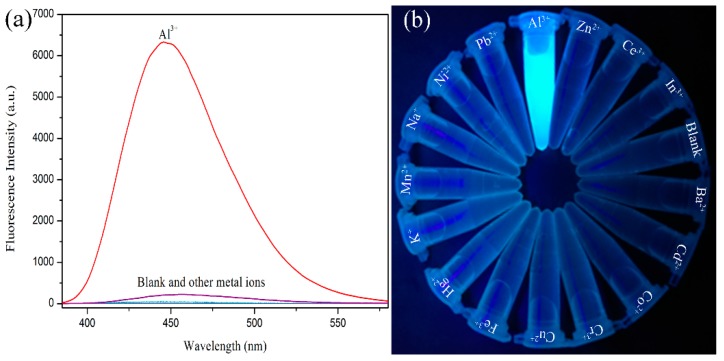
(**a**) Fluorescence spectra of PEGBAB (10 μM) in the presence of various metal ions (2 equiv.) in aqueous solution. (**b**) The photograph of PEGBAB with various metal ions (2 equiv.) in aqueous solution under a 365 nm UV lamp.

The sensing behavior of PEGBAB with different metal ions in pure aqueous solution was further studied by fluorescence measurements. As shown in Figure 1a, the aqueous solution of PEGBAB showed no distinguishable fluorescence response at 447 nm upon excitation at 367 nm. A remarkable enhancement of fluorescence intensity at 447 nm was observed after adding 2 equiv. of Al^3+^ to the PEGBAB solution. However, no obvious fluorescence changes were observed at 447 nm in the solutions of PEGBAB with 2 equiv. of other metal ions. The quantum yields of PEGBAB solutions with 2 equiv. of Al^3+^ was calculated to be 0.267 using quinine sulfate as the standard (Φ_F_ = 0.546 in 0.5 M H_2_SO_4_ solution) [53]. Meanwhile, after adding Al^3+^ to the PEGBAB solution, the noticeable fluorescence change from non-fluorescence to strong bright cyan fluorescence could be observed immediately by naked eye under a 365 nm UV lamp (Figure 1b). The addition of other metal ions could not induce obvious fluorescence color changes of the solutions under a 365 nm UV lamp. The remarkable enhancement of the fluorescence emission intensity and the significant change of the fluorescence color of PEGBAB solution in the presence of Al^3+^ clearly indicates that PEGBAB is an efficient turn-on fluorescent chemosensor for the exclusive detection of Al^3+^ in 100% aqueous solution.

**Figure 2:**
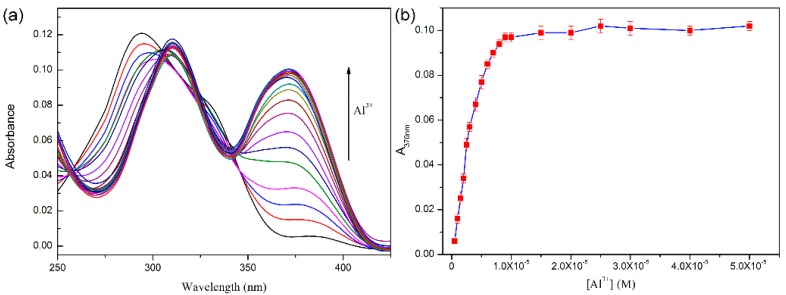
(**a**) UV-Vis absorption spectra of PEGBAB (10 μM) with the increasing concentration of Al^3+^ (0–50 μM) in aqueous solution. (**b**) Plot of absorption intensity at 370 nm against the concentration of Al^3+^.

In order to understand the binding behavior of PEGBAB with Al^3+^, the UV-Vis titration experiment of PEGBAB with a different concentration of Al^3+^ was conducted in pure aqueous solution. As shown in Figure 2, free PEGBAB in aqueous solution showed an absorbance band centered at 295 nm, and a new absorption band appeared at 370 nm after the addition of Al^3+^. Upon the addition of increasing concentrations of Al^3+^ ions into the PEGBAB solution, the absorption intensity at 370 nm increased gradually. Simultaneously, two isosbestic points at around 304 and 342 nm were observed, which implied the transfer of free PEGBAB to a new stable complex PEGBAB-Al^3+^. As shown in the inset of Figure 2, the changes in the absorption intensity at 370 nm became steady when about 1 equiv. of Al^3+^ was added. According to the linear Benesi-Hildebrand expression, the measured absorbance [1/(A − A_0_)] at 370 nm varied as a function of 1/[Al^3+^] in a linear relationship, and the association constant of PEGBAB with Al^3+^ in water was found to be 1.28 × 10^5^ M^−1^ (Figure S7).

**Figure 3:**
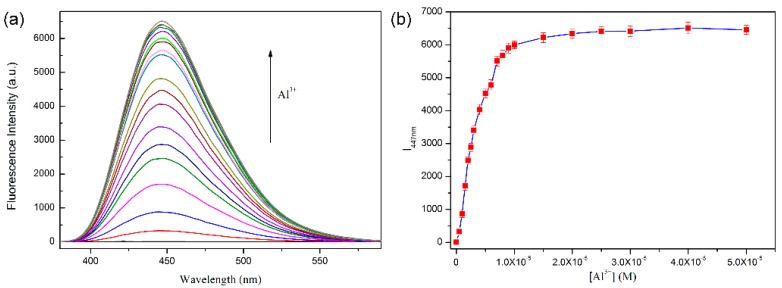
(**a**) Fluorescence spectra of PEGBAB (10 μM) with the increasing concentration of Al^3+^ (0–50 μM) in aqueous solution. (**b**) Plot of fluorescence intensity at 447 nm against the concentration of Al^3+^.

**Scheme 2:**
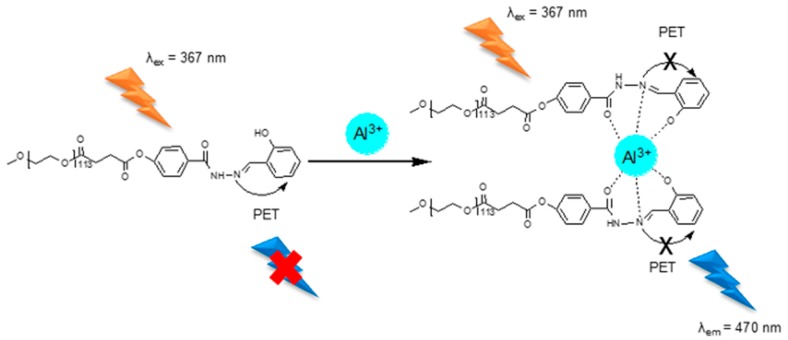
The proposed sensing mechanism of PEGBAB for the detection of Al^3+^.

To further evaluate the binding behavior of PEGBAB with Al^3+^, the fluorescence titration experiments were also carried out. As shown in Figure 3, the free PEGBAB showed negligible fluorescence in pure aqueous solution, and the fluorescence intensity centered at 447 nm enhanced dramatically with the increasing concentration of Al^3+^. After the addition of about 1 equiv. of Al^3+^, the fluorescence intensity of the aqueous solution also reached a stable state and did not change obviously even with the higher concentration of Al^3+^. Under a 365 nm UV lamp, the gradual fluorescence change of PEGBAB solutions from non-fluorescence to bright cyan fluorescence was observed, and the bright intensity of the solution also did not change obviously upon the excess addition of Al^3+^ (Figure S8). Owing to the C=N isomerization and the photo-induced electron transfer (PET) of the lone pair electrons from imine to the benzene ring in the excited state [12,54,55,56], free PEGBAB showed no fluorescence emission. Upon the addition of Al^3+^, a stable chelation of Al^3+^ to PEGBAB inhibited C=N isomerization and PET process efficiently, resulting in a significant fluorescence emission. The binding stoichiometry of the complex between PEGBAB and Al^3+^ was confirmed to be 2:1 by Job’s plot analysis (Figure S9). Based on the above results, the proposed sensing mechanism of PEGBAB for the detection of Al^3+^ was shown in Scheme 2. The association constant of PEGBAB with Al^3+^ calculated from the Benesi-Hildebrand equation was found to be 1.18 × 10^5^ M^−1^ based on the fluorescence titration (Figure S10), which was quite close to that of the UV-Vis titration experiment. Furthermore, the detection limit of PEGBAB for Al^3+^ was calculated to be 4.05 × 10^−9^ M according to 3σ/k, where σ is the standard deviation of the blank solution (fluorescence intensity at 447 nm of PEGBAB in pure water), and k is the slope of the calibration curve between fluorescence intensity and Al^3+^ concentration (Figure S11). The detection limit was much lower than the WHO’s recommended value of 7.41 μM in drinking water [57]. PEGBAB exhibited a lower detection limit for Al^3+^ than our previously reported chemosensor PEGBHB [51], which has the opposite structure compared with PEGBAB. As shown in Table 1, the detection limit of PEGBAB for Al^3+^ is comparable or better than other reported chemosensors. Moreover, PEGBAB has outstanding water solubility and can be used to detect Al^3+^ in pure water without the addition of any organic cosolvents.

### 3.3. Competition Experiment 

**Figure 4:**
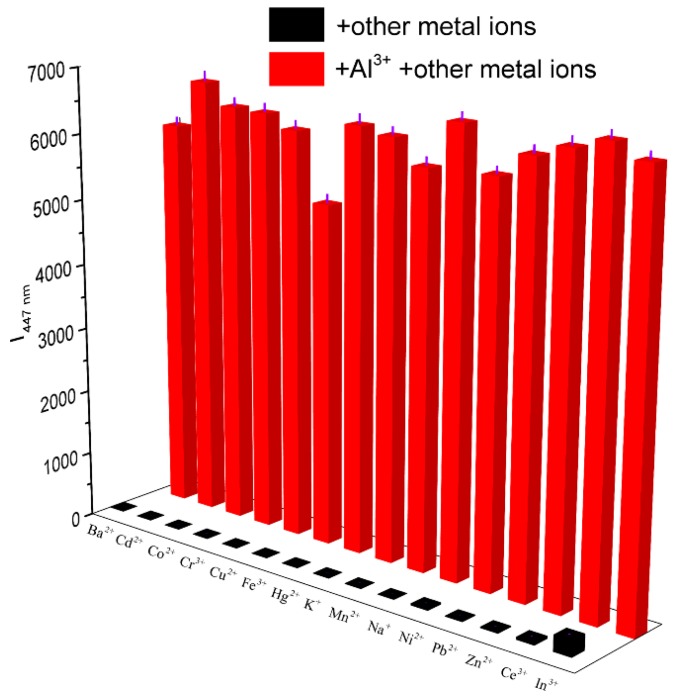
Bar graph of fluorescence intensity at 447 nm of PEGBAB (10 μM) with and without Al^3+^ (2 equiv.) in the presence of other competitive metal ions (2 equiv.) in aqueous solutions.

To explore the applicability of PEGBAB as a selective chemosensor for Al^3+^, competition experiments of PEGBAB to Al^3+^ were conducted in the presence of other competitive metal ions (Ba^2+^, Ce^3+^, Cd^2+^, Co^2+^, Cr^3+^, Cu^2+^, Fe^3+^, Hg^2+^, In^3+^, K^+^, Mn^2+^, Na^+^, Ni^2+^, Pb^2+^ and Zn^2+^). As shown in Figure 4, the fluorescence intensity of PEGBAB to Al^3+^ was not significantly influenced by coexisting competitive metal ions. It could be concluded that the existence of other metal ions did not influence the chelation of Al^3+^ with PEGBAB. Thus, PEGBAB can be employed as a highly selective fluorescent chemosensor for Al^3+^ over other competitive metal ions in pure aqueous solution.

### 3.4. Effect of pH and Temperature

**Figure 5:**
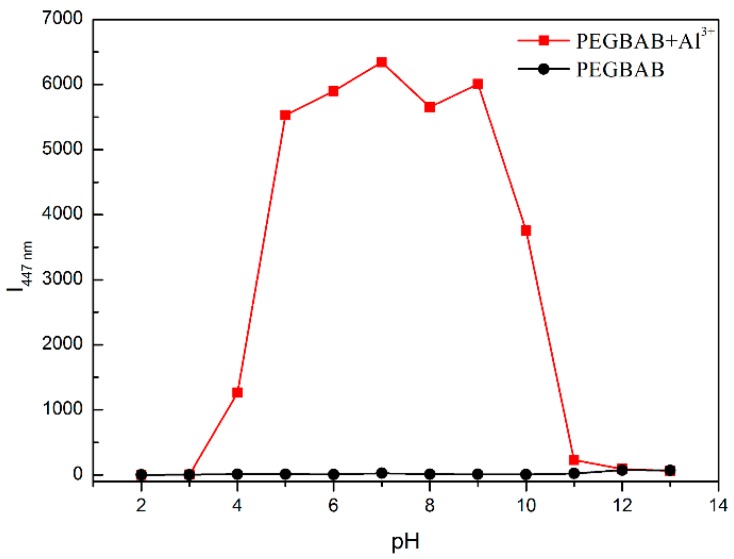
Effect of pH on the fluorescence intensity at 447 nm of PEGBAB (10 μM) in aqueous solutions in the absence and presence of Al^3+^ (2 equiv.).

Generally, the pH value of the solutions could affect the sensing behavior of most fluorescent chemosensors in practical applications, and so the pH effect on the fluorescence responses of PEGBAB with and without the addition of Al^3+^ was investigated in aqueous solutions with pH values ranging from 2 to 13. As shown in Figure 5, the aqueous solutions of free PEGBAB showed negligible changes in the fluorescence intensity at 447 nm within the pH range of 2–13. However, after adding 2 equiv. of Al^3+^, different fluorescence responses of PEGBAB solutions were observed at a different pH range. Because the protonation of the Schiff-based group in PEGBAB inhibited the binding of Al^3+^ to PEGBAB at strong acidic conditions, the fluorescence intensity of the solution was quite weak when the pH was lower than 3. With the decrease of the acidity, the fluorescence intensity increased rapidly from pH 3 to 5. When the pH value was above 9, the fluorescence intensity of the solution went down dramatically due to the formation of Al(OH)_3_ or Al(OH)_4_^−^ at strong alkaline conditions. Therefore, PEGBAB is an efficient turn-on fluorescent chemosensor for the detection Al^3+^ in the pH range from 5 to 10 and can be a suitable candidate for environmental and biological applications. Furthermore, a wide test temperature range is also important for practical applications. The detection stability of PEGBAB for Al^3+^ was studied in the temperature range of 20–80 °C. As shown in Figure S12, the fluorescence intensities of PEGBAB solutions upon the addition of 2 equiv. of Al^3+^ were quite stable within the temperature range of 20–80 °C, meaning that PEGBAB exhibited high sensing stability for Al^3+^ in the wide temperature range.

### 3.5. Reversibility Study

**Figure 6:**
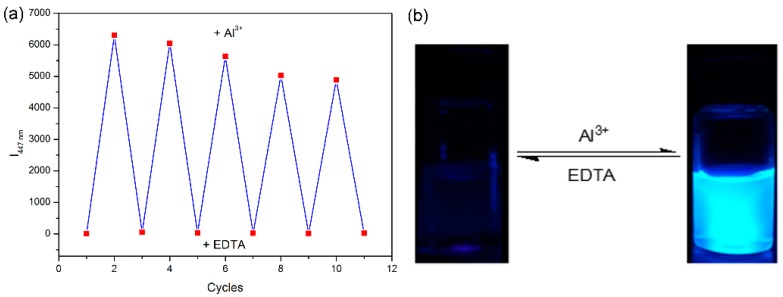
(**a**) Reversible switching cycles of fluorescence intensity at 447 nm by alternate addition of Al^3+^ (1 equiv.) and EDTA (1 equiv.) in aqueous solution of PEGBAB (10 μM). (**b**) Reversible fluorescence changes of PEGBAB (10 μM) in aqueous solution by alternate addition of Al^3+^ (1 equiv.) and EDTA (1 equiv.).

The reversibility of fluorescence response is one of the significant features of an excellent fluorescent chemosensor for practical application. The reversible binding of Al^3+^ to PEGBAB was investigated by fluorescence titration of PEGBAB solution upon the alternate addition of Al^3+^ and EDTA. As illustrated in Figure S13, after adding 1 equiv. of EDTA to the PEGBAB-Al^3+^ solution, the fluorescence of the solution was quenched and the fluorescent intensity at 447 nm returned to the initial intensity of free PEGBAB solution. After the addition of 1 equiv. of Al^3+^ again, the fluorescence of the solution was recovered. Furthermore, upon the alternate addition of Al^3+^ and EDTA into PEGBAB solution, the fluorescence switching response could be repeated for several cycles (Figure 6). The decrease of the fluorescence intensity might be caused by the excess EDTA that remained in the solution, which hindered the effective complexation of Al^3+^ with PEGBAB. These results suggest that PEGBAB can be used as a reversible fluorescent chemosensor for the detection of Al^3+^ in 100% aqueous solution.

### 3.6. Construction of Logic Gate

**Figure 7:**
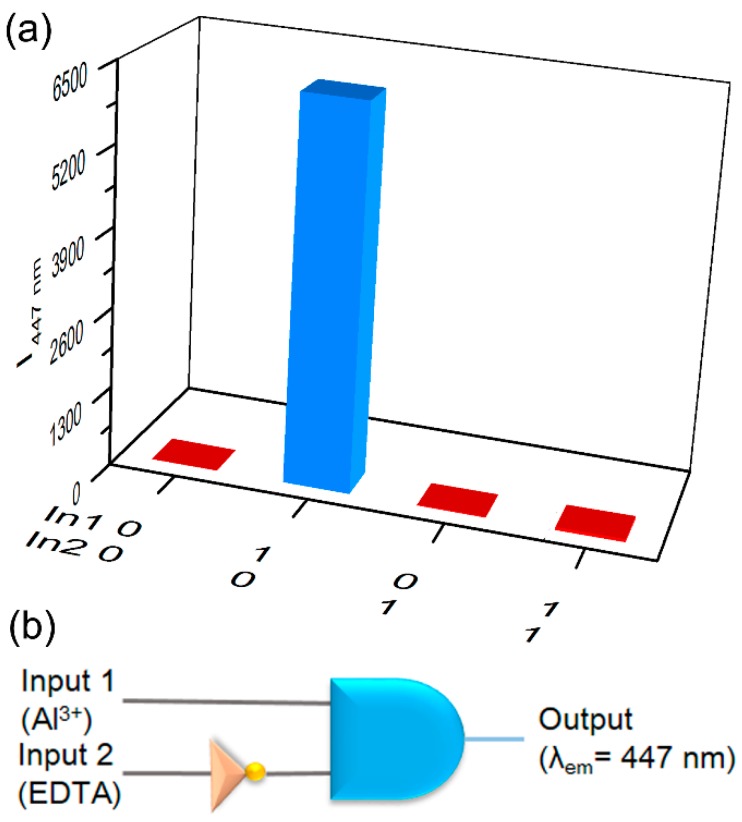
(**a**) The fluorescence intensity of PEGBAB at 447 nm in the presence of four different inputs. (**b**) The circuit diagram for the INHIBIT logic gate.

Since the seminal work about the two-input molecular logic gate reported by de Silva [65], the construction of molecular logic gate based on fluorescent chemosensors has attracted significant interests [31,35,62,66,67,68]. Due to the fluorescence response of PEGBAB to Al^3+^ and EDTA, an INHIBIT type molecular logic gate could be constructed with Al^3+^ (In 1) and EDTA (In 2) as two chemical inputs and the fluorescence intensity at 447 nm as output. The presence of Al^3+^ and EDTA was defined as “1”, and the absence of these inputs was defined as “0” (Table 2). For the output, turn-on and turn-off of the fluorescence were defined as “0” and “1”, respectively. As shown in Figure 7a, only when the individual input of Al^3+^ was present, output “1” was obtained because the PEGBAB solution showed a prominent turn-on fluorescence response at 447 nm. In other input combinations, the output signal was “0”, implying turn-off of the fluorescence. The corresponding circuit diagram for the INHIBIT logic gate is shown in Figure 7b.

### 3.7. Application as Test Strips

**Figure 8:**
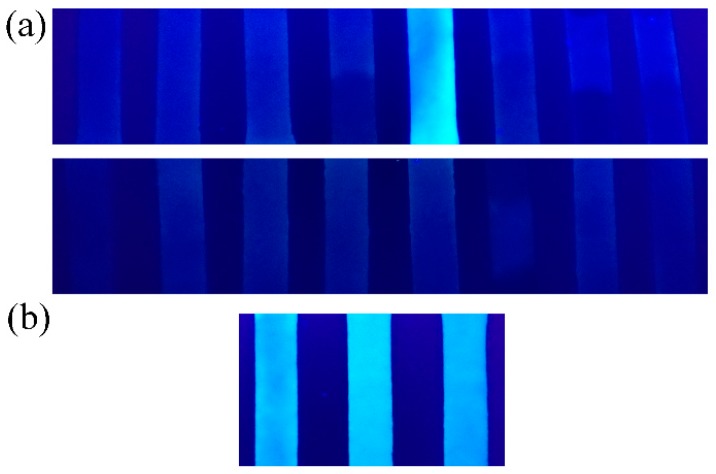
Photographs of test strips under a 365 nm UV lamp: (**a**) after being dipped into aqueous solutions of different metal ions (100 μM) (from left to right, up: Ba^2+^, Ce^3+^, Cd^2+^, Co^2+^, Al^3+^, Cr^3+^, Cu^2+^ and Fe^3+^; down: Hg^2+^, In^3+^, K^+^, Mn^2+^, Na^+^, Ni^2+^, Pb^2+^ and Zn^2+^); (**b**) after being dipped into different solutions of Al^3+^ (100 μM) (from left to right: deionized water, tap water and river water).

Test strip method has been proved to be an efficient strategy to evaluate the practical application of fluorescent chemosensors [69,70,71]. The test strips coated with PEGBAB were prepared by immersing the filter papers into the methanol solution of PEGBAB and then drying in air. After being dipped in a 10^−4^ M aqueous solution of Al^3+^, the test strip exhibited strong bright cyan fluorescence under a 365 nm UV lamp, and the bright intensity of the test strips increased with the increasing concentrations of Al^3+^ (Figure S14). The test strips were utilized to recognize Al^3+^ over various metal ions and in different water environments. As shown in Figure 8a, when test strips were dipped into aqueous solutions of various metal ions, only the test strip with Al^3+^ showed a significant visible color change from non-fluorescence to strong bright cyan fluorescence under a 365 nm UV lamp. The competition experiments of test strips to Al^3+^ were also carried out in the presence of other competitive metal ions, and the bright fluorescence of the test strip to Al^3+^ was unaffected by other coexisting metal ions (Figure S15). Though PEGBAB in tap water and river water exhibited fluorescence emission compared with PEGBAB in deionized water, the fluorescence intensities were both very weak (Figure S16), which had no influence on the practical application of test strips. Upon dipping the test strips into different Al^3+^ solutions in deionized water, tap water and river water, all of these test strips exhibited distinct bright cyan fluorescence under a 365 nm UV lamp (Figure 8b). Furthermore, the temperature effect on the application of test strips was also investigated within the temperature range of 20–80 °C. As shown in Figure S17, the test strips all showed distinct bright cyan fluorescence from 20 °C to 80 °C under a 365 nm UV lamp. After being stored in air for 7 days, the test strip could still exhibit bright cyan fluorescence after being dipped into Al^3+^ solution (Figure S18), meaning that the prepared test strips were quite stable. Therefore, test strips coated with PEGBAB can be employed to on-site detect Al^3+^ in a real water environment.

## 4. Conclusions

In this paper, we have successfully developed a novel water-soluble polymer-based fluorescent chemosensor PEGBAB by incorporating salicylidene Schiff base derivative into the hydrophilic polymer PEG. PEGBAB exhibited excellent sensitivity and selectivity to Al^3+^ over other competitive metal ions in pure aqueous solutions. PEGBAB was stable over the investigated pH range of 2–13 and showed good fluorescence sensing ability to Al^3+^ in the pH range from 5 to 10. The detection limit of PEGBAB for Al^3+^ was as low as 4.05 × 10^−9^ M. The fluorescence quenching of PEGBAB-Al^3+^ complex by EDTA indicated that PEGBAB could serve as a reversible fluorescent chemosensor. The INHIBIT logic gate has been constructed on the basis of the fluorescence response of PEGBAB to Al^3+^ and EDTA. More importantly, test strips coated with PEGBAB were fabricated facilely, which could serve as efficient and convenient test kits for on-site detection of Al^3+^ in a real water environment. This work provides an efficient strategy for the development of Schiff base-based polymer chemosensors for highly selective and sensitive detection of metal ions in pure aqueous solution.

## Figures and Tables

**Table 1:** Comparison of PEGBAB with some reported chemosensors for the detection of Al^3+^.

Chemosensor	Association Constant (M^−1^)	Detection Limit (M)	Testing Media	Refs.
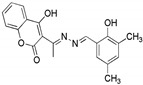	8.5 × 10^5^	1.05 × 10^−8^	DMSO/H_2_O (1:5, *v*/*v*)	[35]
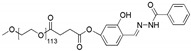	1.30 × 10^5^	9.67 × 10^−9^	100% H_2_O	[51]
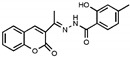	3.21 × 10^6^	6.7 × 10^−6^	DMF/water (9:1, *v*/*v*)	[54]
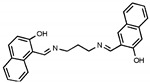	7.62 × 10^6^	3.7 × 10^−7^	EtOH/H_2_O (1:1, *v*/*v*)	[58]
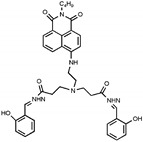	5.49 × 10^4^	1.78 × 10^−7^	MeOH/H_2_O (1:1, *v*/*v*)	[59]
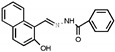	4.8 × 10^5^	8.87 × 10^−7^	CH_3_CN/H_2_O (1:1, *v*/*v*)	[60]
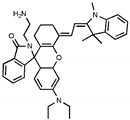	9.87 × 10^4^	3.0 × 10^−8^	EtOH/H_2_O (1:99, *v*/*v*)	[61]
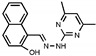	1.9 × 10^4^	2.78 × 10^−6^	EtOH	[62]
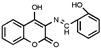	(1.06 ± 0.2) × 10^4^	1.34 × 10^−6^	MeOH/H_2_O (9:1, *v*/*v*)	[63]
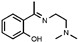	2.1 × 10^4^	4.32 × 10^−6^	MeOH/H_2_O (9:1, *v*/*v*)	[64]
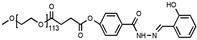	1.28 × 10^5^ (UV-Vis) 1.18 × 10^5^ (fluorescence)	4.05 × 10^−9^	100% H_2_O	This work

**Table 2:** Truth table for the INHIBIT logic gate.

Input 1 (Al^3+^)	Input 2 (EDTA)	Output (λ_em_ = 447 nm)
0	0	0
1	0	1
0	1	0
1	1	0

## Supplementary Materials

The following are available online at https://www.mdpi.com/2073-4360/11/4/573/s1, Figure S1: ^1^H NMR spectrum of BAB in DMSO-*d*_6_; Figure S2: ^13^C NMR spectrum of BAB in DMSO-*d*_6_; Figure S3: ESI-MS spectrum of BAB; Figure S4: ^1^H NMR spectrum of PEGBAB in CDCl_3_; Figure S5: ^13^C NMR spectrum of PEGBAB in CDCl_3_; Figure S6: UV-Vis absorption spectra of PEGBAB (10 μM) with 2 equiv. of various metal ions in pure aqueous solutions; Figure S7: Benesi-Hildebrand plot of PEGBAB (10 μM) with Al^3+^ from UV-Vis titration profile for determination of association constant; Figure S8: The photograph of PEGBAB (10 μM) in aqueous solution with different concentrations of Al^3+^ (from left to right: 0, 0.1, 0.25, 0.5, 1, 2, 5 equiv.) under a 365 nm UV lamp; Figure S9: Job’s plot of PEGBAB and Al^3+^ in aqueous solution. The total concentration of PEGBAB and Al^3+^ is 10 μM; Figure S10: Benesi-Hildebrand plot of PEGBAB (10 μM) with Al^3+^ from fluorescence titration profile for determination of association constant; Figure S11: The linear of fluorescence intensity and concentration of Al^3+^ for the determination of limit of detection; Figure S12: Effect of temperature on the fluorescence intensity at 447 nm of PEGBAB (10 μM) in aqueous solutions upon addition of 2 equiv. of Al^3+^; Figure S13: Fluorescence spectra of PEGBAB (10 μM) in aqueous solution upon alternate addition of Al^3+^ (1 equiv.) and EDTA (1 equiv.); Figure S14: Photographs of test strips after being dipped into Al^3+^ solutions with different concentrations under a 365 nm UV lamp (from left to right: 10^−6^ M, 10^−5^ M, 10^−4^ M, 10^−3^ M, 10^−2^ M); Figure S15: Photographs of test strips after being dipped into aqueous solutions of Al^3+^ (100 μM) mixed with different metal ions (100 μM) under a 365 nm UV lamp (from left to right, up: Al^3+^, Ba^2+^, Ce^3+^, Cd^2+^, Co^2+^, Cr^3+^, Cu^2+^ and Fe^3+^; down: Hg^2+^, In^3+^, K^+^, Mn^2+^, Na^+^, Ni^2+^, Pb^2+^ and Zn^2+^); Figure S16: Fluorescence spectra of PEGBAB (10 μM) in different aqueous solution; Figure S17: Photographs of test strips after being dipped into Al^3+^ solutions (100 μM) at different temperature under a 365 nm UV lamp (from left to right: 20 °C, 30 °C, 40 °C, 50 °C, 60 °C, 70 °C, 80 °C); Figure S18: Photographs of test strips after being dipped into Al^3+^ solutions (100 μM) under a 365 nm UV lamp: (a) stored in air for 1 day; (b) stored in air for 7 days.
