# Supplementary material for: A Highly Selective Turn-on and Reversible Fluorescent Chemosensor for Al3+ Detection Based on Novel Salicylidene Schiff Base-Terminated PEG in Pure Aqueous Solution
Source: Polymers (Basel). 2019 Mar 27;11(4):573. doi: 10.3390/polym11040573 (PMC6523932; doi:10.3390/polym11040573)
Supplement: Supplementary file 1 [file polymers-11-00573-s001.pdf]

A highly selective turn-on and reversible fluorescent chemosensor for Al^{3+} detection based on novel salicylidene Schiff base-terminated PEG in pure aqueous solution

Liping Bai, Yuhang Xu, Guang Li*, Shuhui Tian, Leixuan Li, Farong Tao, Aixia Deng, Shuangshuang Wang and Liping Wang*

[1] School of Materials Science and Engineering, Liaocheng University, Liaocheng 252059, China

* Correspondence: lglzsd@126.com (G.L.); wangliping5@163.com (L.W.); Tel: +86-635-823-0919

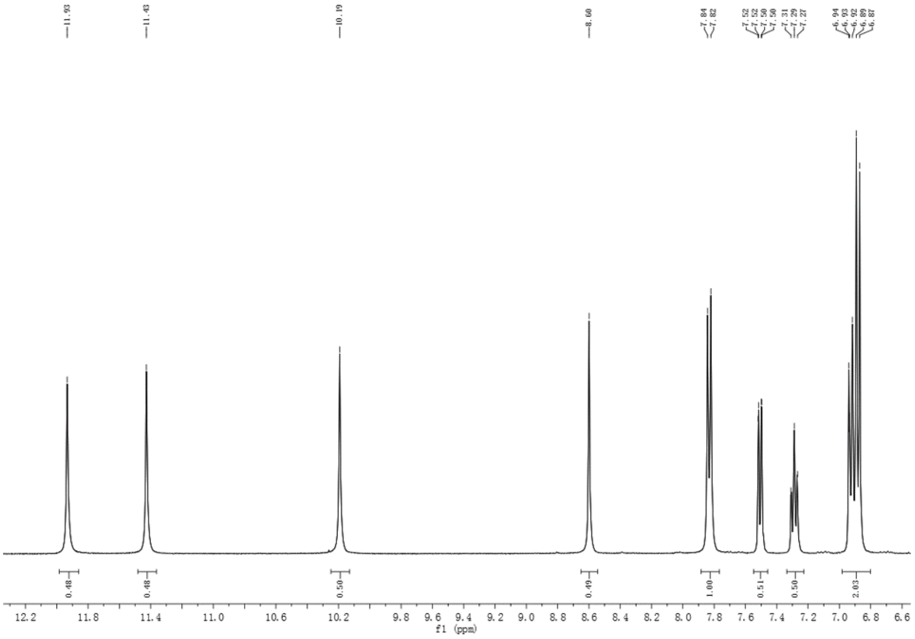

Figure S1. ^{1}H NMR spectrum of BAB in DMSO-d_6.

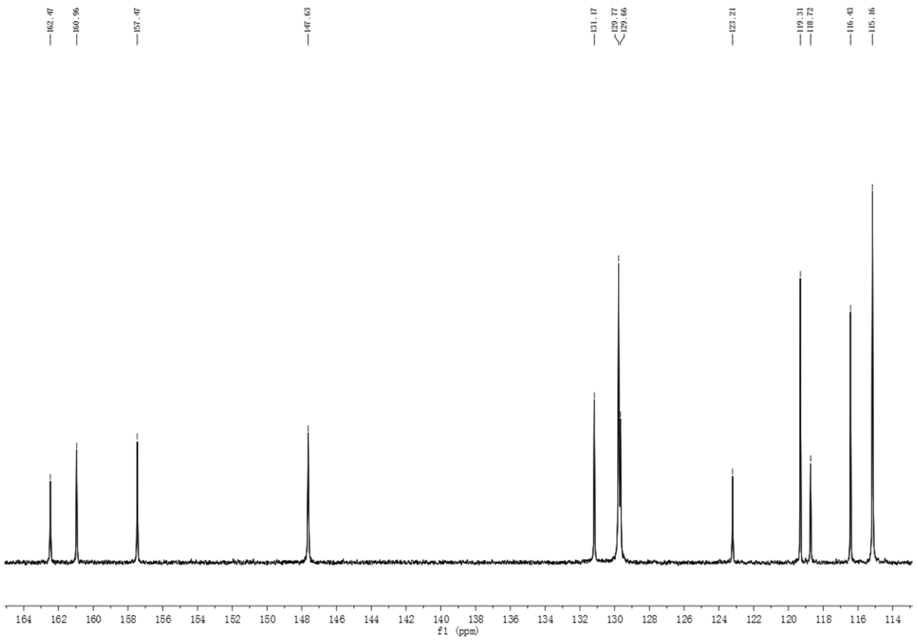

Figure S2. ^{13}C NMR spectrum of BAB in DMSO-d_6.

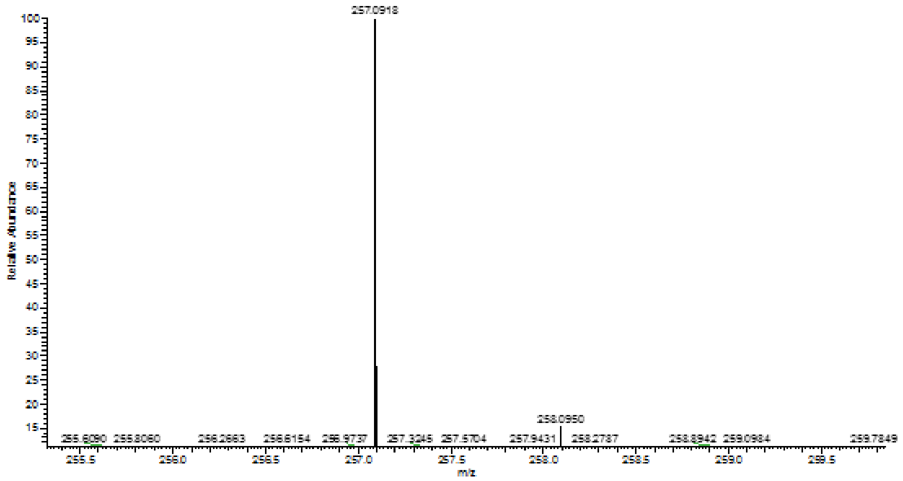

Figure S3. ESI-MS spectrum of BAB.

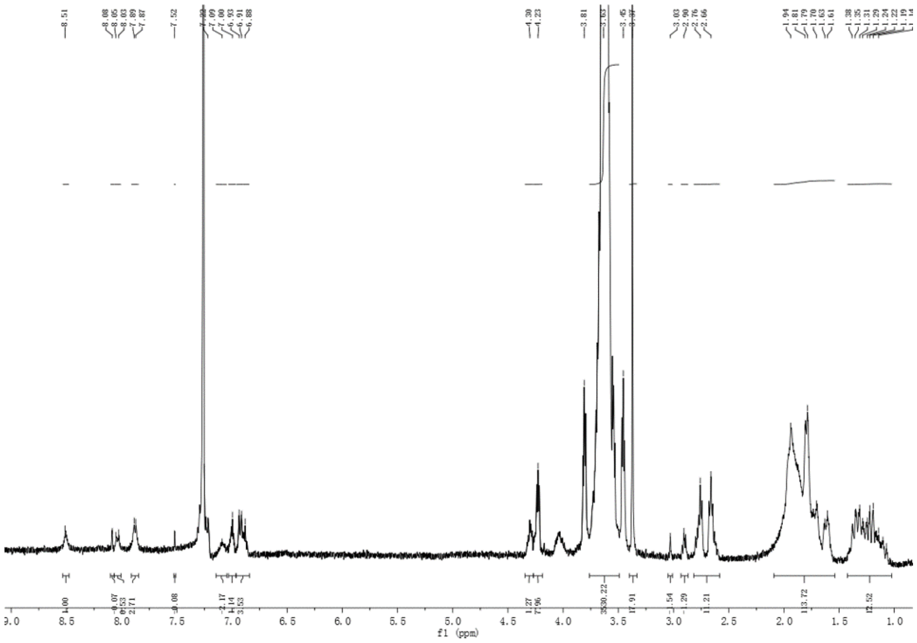

Figure S4. ^{1}H NMR spectrum of PEGBAB in $CDCl_3$.

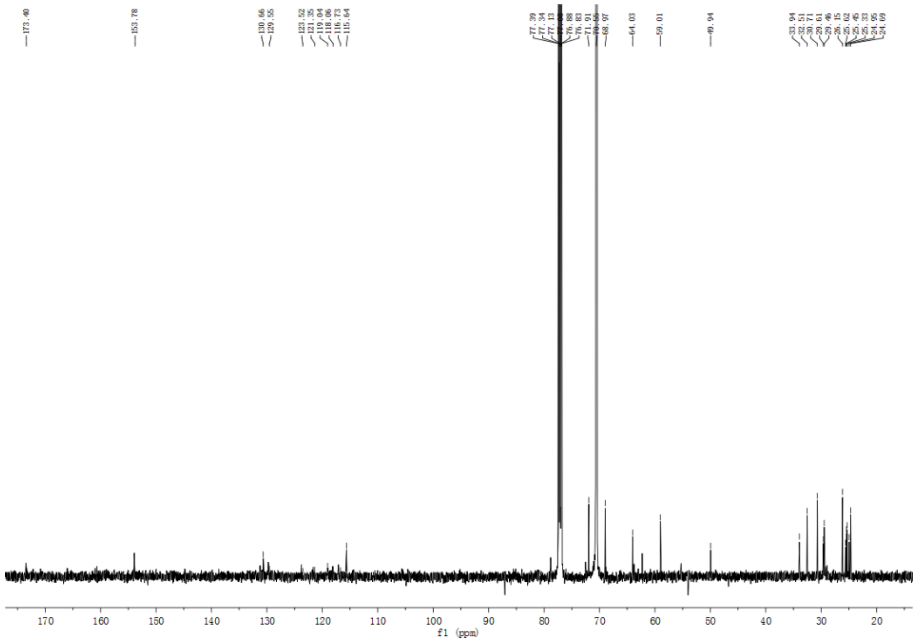

Figure S5. ^{13}C NMR spectrum of PEGBAB in $CDCl_3$.

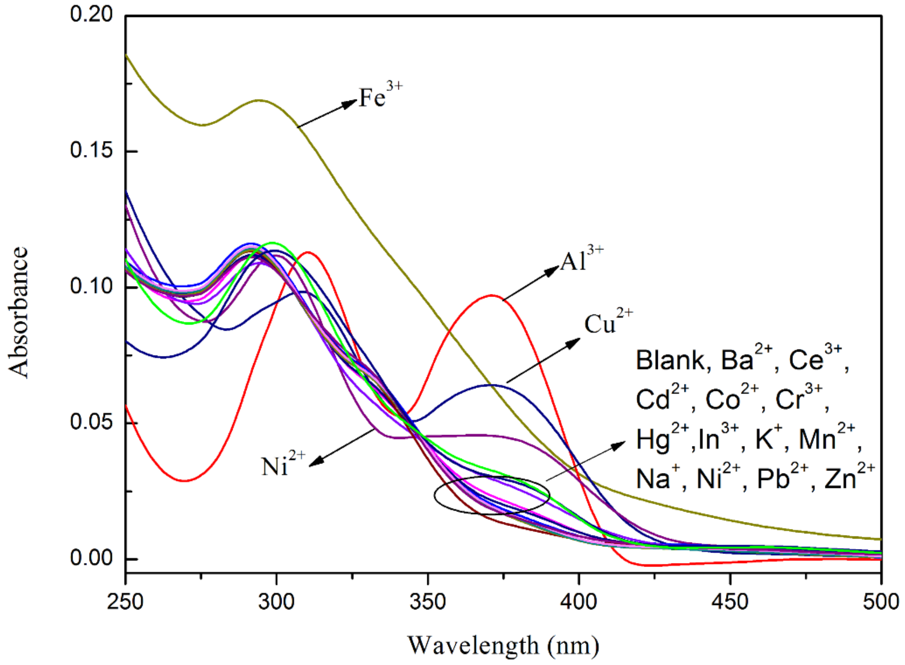

Figure S6. UV-Vis absorption spectra of PEGBAB (10 μM) with 2 equiv. of various metal ions in pure aqueous solutions.

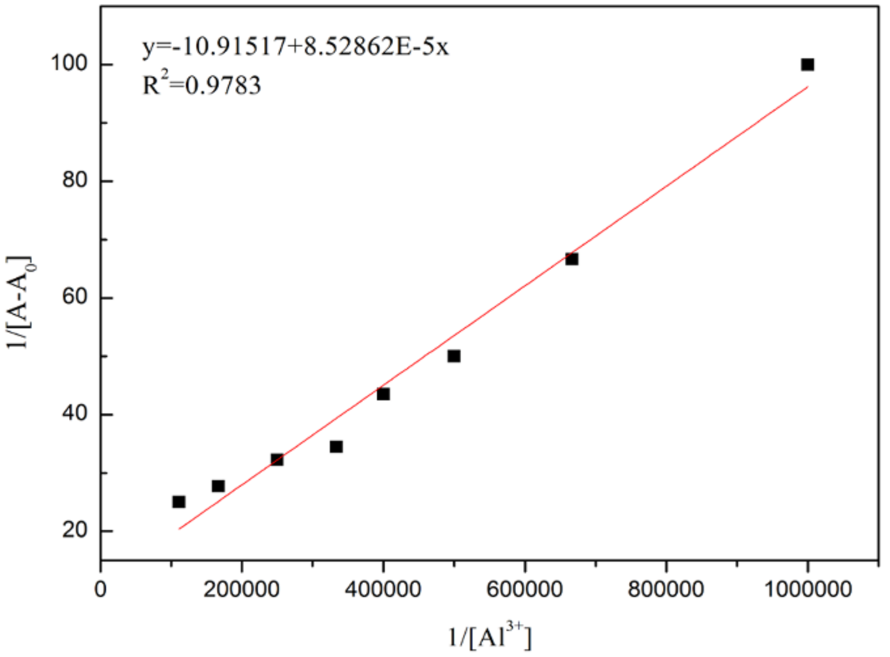

Figure S7. Benesi-Hildebrand plot of PEGBAB (10 μM) with Al^{3+} from UV-Vis titration profile for determination of association constant.

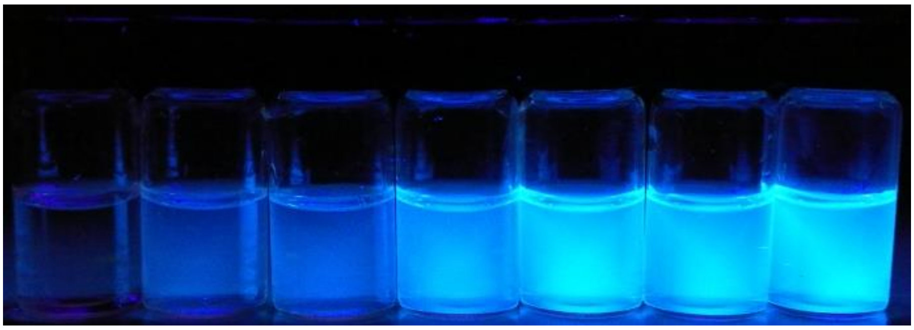

Figure S8. The photograph of PEGBAB (10 μM) in aqueous solution with different concentrations of Al^{3+} (from left to right: 0, 0.1, 0.25, 0.5, 1, 2, 5 equiv.) under a 365 nm UV lamp.

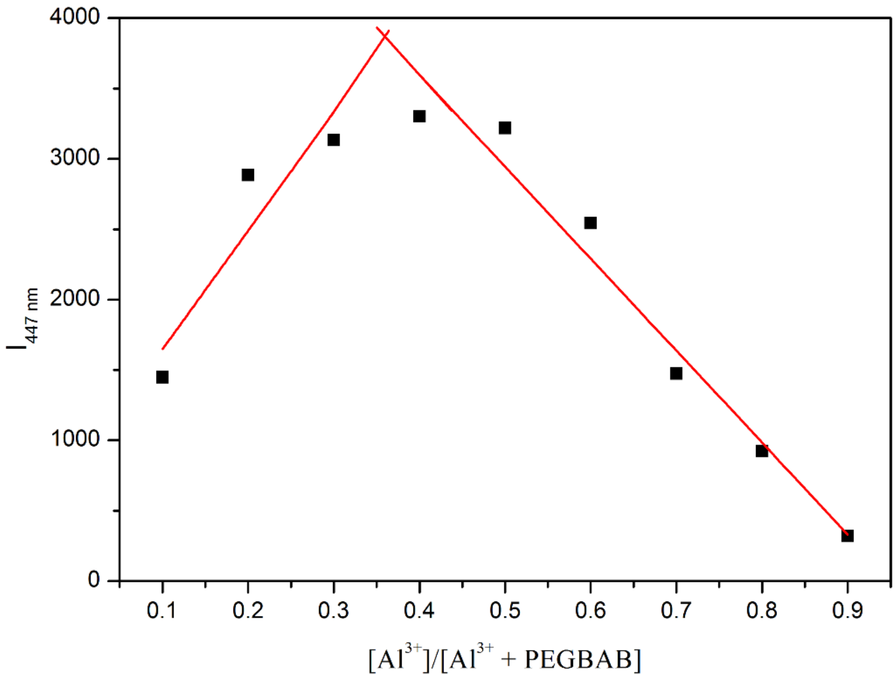

Figure S9. Job's plot of PEGBAB and Al^{3+} in aqueous solution. The total concentration of PEGBAB and Al^{3+} is 10 μM.

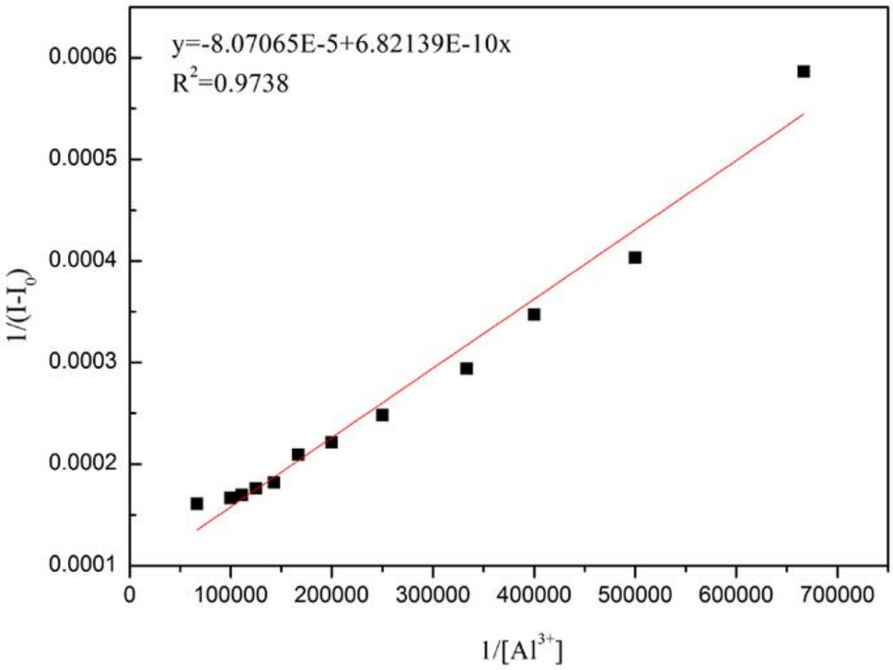

Figure S10. Benesi-Hildebrand plot of PEGBAB (10 μM) with Al^{3+} from fluorescence titration profile for determination of association constant.

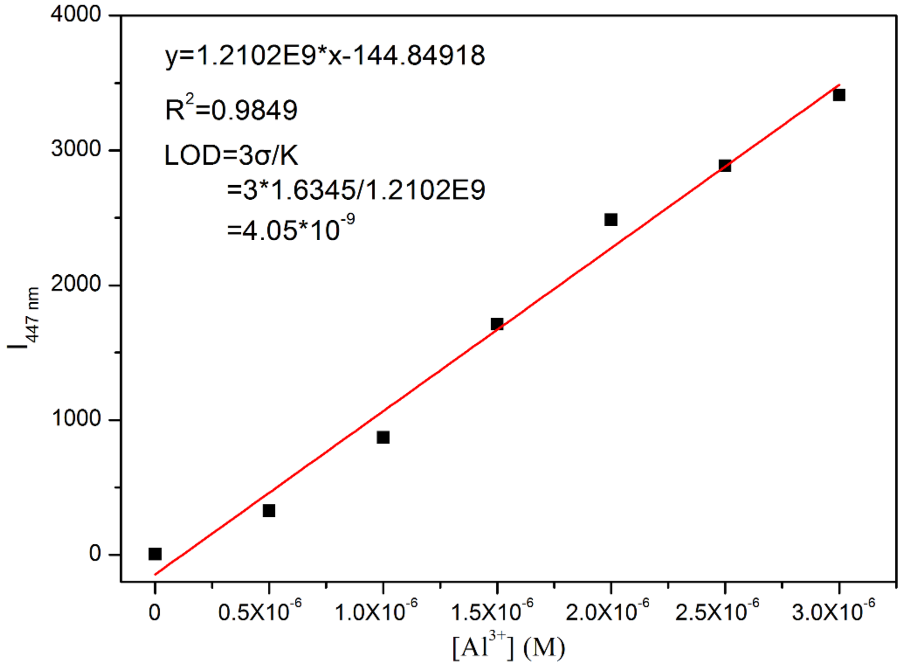

Figure S11. The linear of fluorescence intensity and concentration of Al^{3+} for the determination of limit of detection.

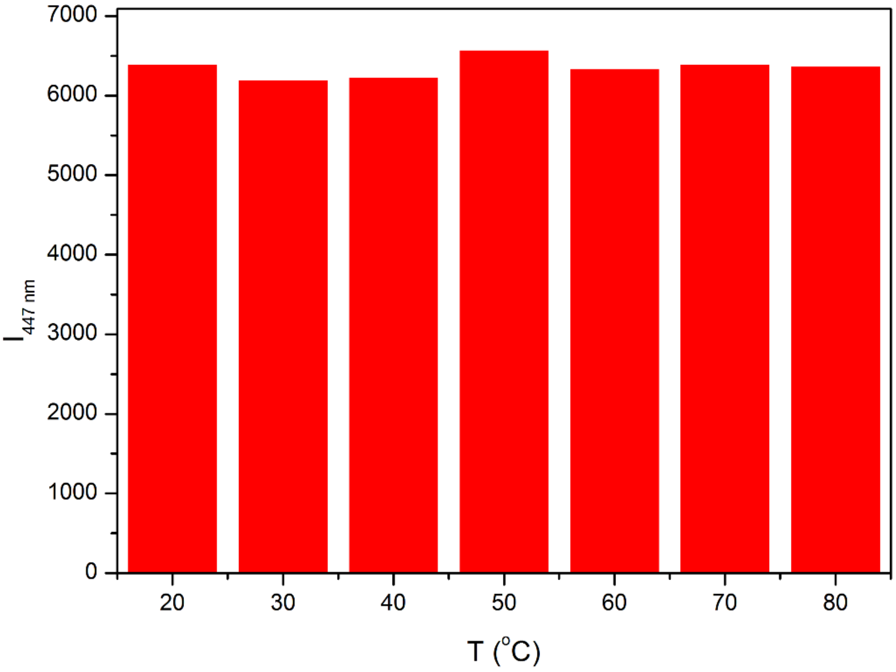

Figure S12. Effect of temperature on the fluorescence intensity at 447 nm of PEGBAB (10 μM) in aqueous solutions upon addition of 2 equiv. of Al^{3+}.

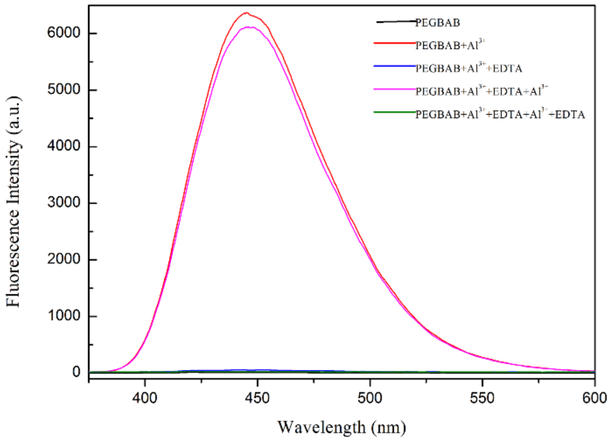

Figure S13. Fluorescence spectra of PEGBAB (10 μM) in aqueous solution upon alternate addition of Al^{3+} (1 equiv.) and EDTA (1 equiv.).

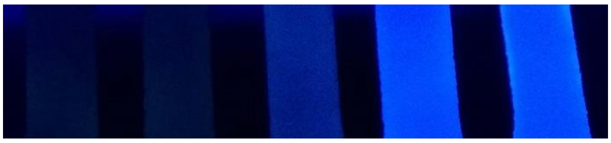

Figure S14. Photographs of test strips after being dipped into Al^{3+} solutions with different concentrations under a 365 nm UV lamp (from left to right: 10^{-6} M, 10^{-5} M, 10^{-4} M, 10^{-3} M, 10^{-2} M).

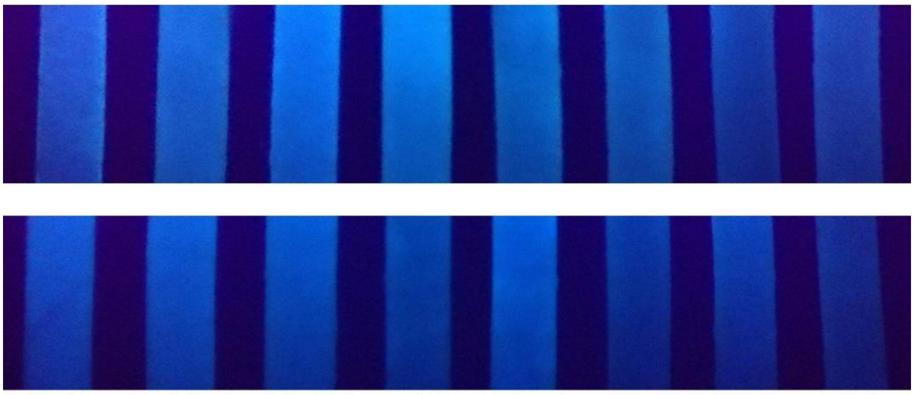

Figure S15. Photographs of test strips after being dipped into aqueous solutions of Al^{3+} (100 μM) mixed with different metal ions (100 μM) under a 365 nm UV lamp (from left to right, up: Al^{3+}, Ba^{2+}, Ce^{3+}, Cd^{2+}, Co^{2+}, Cr^{3+}, Cu^{2+} and Fe^{3+}; down: Hg^{2+}, In^{3+}, K^{+}, Mn^{2+}, Na^{+}, Ni^{2+}, Pb^{2+} and Zn^{2+}).

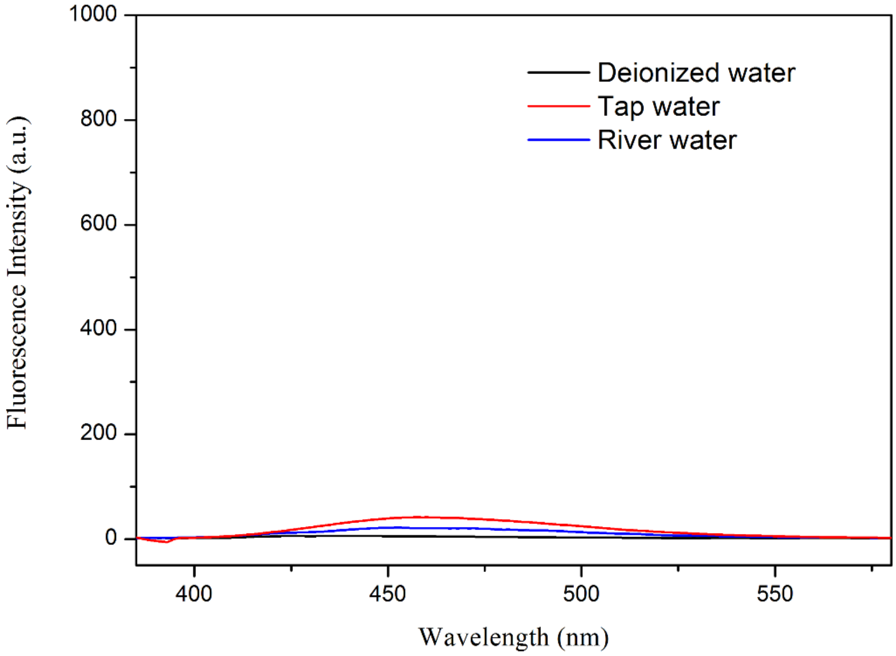

Figure S16. Fluorescence spectra of PEGBAB (10 μM) in different aqueous solution.

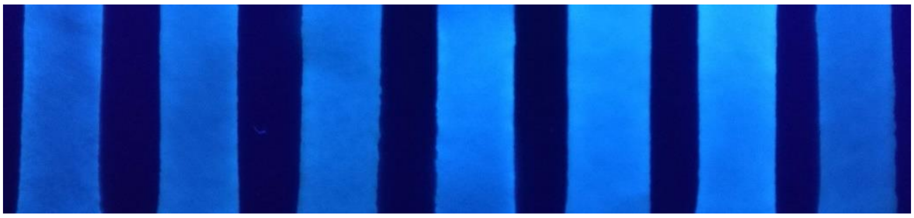

Figure S17. Photographs of test strips after being dipped into Al^{3+} solutions (100 μM) at different temperature under a 365 nm UV lamp (from left to right: 20 °C, 30 °C, 40 °C, 50 °C, 60 °C, 70 °C, 80 °C).

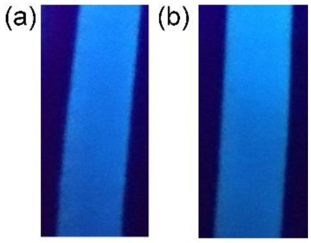

Figure S18. Photographs of test strips after being dipped into Al^{3+} solutions (100 μM) under a 365 nm UV lamp: (a) stored in air for 1 day; (b) stored in air for 7 days.
